# High-density single cell mRNA sequencing to characterize circulating tumor cells in hepatocellular carcinoma

**DOI:** 10.1038/s41598-018-30047-y

**Published:** 2018-08-01

**Authors:** Delia D’Avola, Carlos Villacorta-Martin, Sebastiao N. Martins-Filho, Amanda Craig, Ismail Labgaa, Johann von Felden, Allette Kimaada, Antoinette Bonaccorso, Parissa Tabrizian, Boris M. Hartmann, Robert Sebra, Myron Schwartz, Augusto Villanueva

**Affiliations:** 1 Division of Liver Diseases, Department of Medicine, Liver Cancer Program, Tisch Cancer Institute, Graduate School of Biomedical Sciences, Icahn School of Medicine at Mount Sinai, New York, USA; 2 LiverUnit and Centro de Investigación Biomédica en Red de Enfermedades Hepáticas y Digestivas (CIBEREHD), Clínica Universidad de Navarra, Pamplona, Spain; 3 Departamento de Patologia, Faculdade de Medicina FMUSP, Universidade de Sao Paulo, Sao Paulo, Brazil; 4 Department of Visceral Surgery, Lausanne University Hospital CHUV, Lausanne, Switzerland; 5 Department of Internal Medicine, University Medical Center Hamburg-Eppendorf, Hamburg, Germany; 6 Department of Genetics and Genomic Sciences, Icahn School of Medicine at Mount Sinai, New York, USA; 7 Department of Surgery, Icahn School of Medicine at Mount Sinai, New York, USA; 8 Department of Neurology, Icahn School of Medicine at Mount Sinai, New York, USA; 9 Sema4, a Mount Sinai Venture, Stamford, CT USA; 10 Division of Hematology and Medical Oncology, Department of Medicine, Icahn School of Medicine at Mount Sinai, New York, USA

## Abstract

Patients with hepatocellular carcinoma (HCC) release tumor cells to the bloodstream, which can be detected using cell surface markers. Despite numerous reports suggest a direct correlation between the number of circulating tumor cells (CTCs) and poor clinical outcomes, few studies have provided a thorough molecular characterization of CTCs. Due to the limited access to tissue samples in patients at advanced stages of HCC, it is crucial to develop new technologies to identify HCC cancer drivers in routine clinical conditions. Here, we describe a method that sequentially combines image flow cytometry and high density single-cell mRNA sequencing to identify CTCs in HCC patients. Genome wide expression profiling of CTCs using this approach demonstrates CTC heterogeneity and helps detect known oncogenic drivers in HCC such as IGF2. This integrated approach provides a novel tool for biomarker development in HCC using liquid biopsy.

## Introduction

The concept of liquid biopsy, which entails the analysis of tumor components released to the bloodstream (i.e., DNA and tumor cells), has revolutionized oncology^1^. The main advantages of liquid biopsy are: (1) the technique is minimally invasive, which decreases the financial costs and potential complications of tissue biopsies, and (2) it is easy to iterate during patients’ follow-up. Both features are crucial considering how cancer cells adapt to pharmacological pressures, and acquire new molecular alterations not detected at baseline^2^. To account for this, we would be required to perform repeated invasive tissue biopsies upon every treatment, which is difficult to implement in routine clinical conditions. Thus, to develop new biomarkers using liquid biopsy strategies is imperative. This is even more critical in hepatocellular carcinoma (HCC) because these patients are generally diagnosed using imaging techniques^3^, so unlike most solid tumors, access to tissue to conduct biomarker studies in HCC is significantly more difficult. In the United States, liver cancer (including both HCC and intrahepatic cholangiocarcinoma) is currently the fastest growing malignancy both in terms of incidence and mortality^4^.

Most of the studies exploring circulating tumor cells (CTCs) in HCC have shown a direct correlation between higher CTC number and poor clinical outcomes^5^. In most of them, CTC detection relied on the analysis of a small number of surface markers. Indeed, the only FDA-approved device to detect CTCs is based on the expression of the surface marker EPCAM. These methods do not include isolation of CTCs to conduct a higher resolution molecular characterization, which is critical to use CTCs analysis as a proxy to interrogate dominant tumor molecular alterations. This concept was recently shown in a patient with metastatic breast cancer using whole genome sequencing (WGS) in CTCs^6^, which allowed detecting known driver mutations in the CTC compartment. Similarly, WGS of CTCs isolated from a patient with metastatic prostate cancer revealed driver mutations which could be tracked to corresponding tumor tissue^7^.

There are few data on whole-genome transcriptome profiling of CTCs using single-cell technologies^8^. This was due to the relative low throughput of single-cell isolation techniques, which allowed to sequence in parallel relatively low numbers of cells. Considering the rarity of CTCs in the blood (estimated 0–10 CTCs out of 7,000,000 nucleated cells)^9^, extensive enrichment for CTCs was required before single-cell sequencing. Studies using scRNAseq to analyze CTCs leveraged different approaches to isolate CTCs using robotic or microfluidic devices^10,11^. CTCs isolation heavily relies on the identification of specific markers such as EpCAM in prostate cancer or NG2 in melanoma^10,11^. These studies confirm the ability of genome-wide expression analyses of isolated CTCs to detect de-regulation of genes found in tissue counterparts^10^. Other CTC isolation methods such as those based on cell-surface vimentin expression enabled the identification of PD-L1 as a prognostic marker in patients with colorectal or prostate cancer^12^. Alternative approaches favored the use of PCR-based technologies to derive CTC expression signatures analyzing a limited number of genes, as recently shown in HCC^13^. However, there is limited data on whole-genome transcriptomic profiling of CTCs from HCC patients. Recent technological developments including novel single-cell technologies prompted us to conduct a dense transcriptomic profiling of CTCs using a sequential protocol that maximizes CTC detection rate. We provide the proof-of-principle to further develop single-cell sequencing for biomarker development in HCC.

## Results

### A novel pipeline to detect CTCs using whole transcriptome data

**Figure 1:**
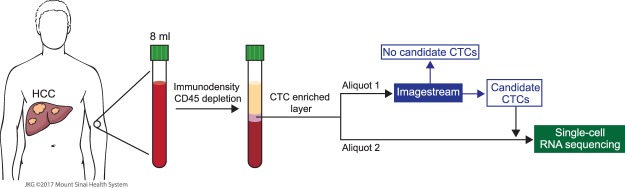
Summary of study design and sample workflow. Illustration by Jill Gregory. Printed with permission from Mount Sinai Health System, licensed under CC BY-ND (https://creativecommons.org/licenses/by-nd/4.0/).

**Figure 2:**
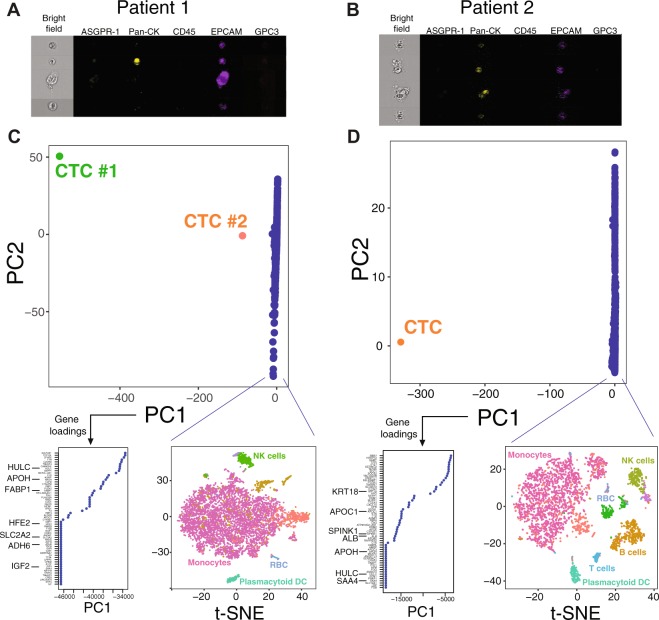
Identification of CTCs using single-cell RNAseq. Top panels show candidate CTCs identified by Imagestream in patients 1 (**A**) and 2 (**B**). (**C**,**D**) PCA plots after outlier-detection normalization for each patient, including the loadings for the top marker genes of PC1, and a t-SNE plot of non-CTCs cells with predicted clusters based on the top-ranked marker genes (RBC: red blood cells, NK: Natural Killer, DC: dendritic cells).

To enable the comprehensive characterization of CTC transcriptome, we developed an analytical pipeline that combines Imaging Flow Cytometry (IFC) and Single-cell RNA sequencing (scRNA-seq). Our protocol uses 8 mL of whole blood and consists of 3 sequential steps: (1) CD45 negative enrichment, (2) selection of patients using IFC, and (3) scRNAseq using 10X Genomics Chromium® (Fig. 1). This enables mRNA sequencing in up to 10,000 cells with a capture efficiency up to 65% for high-throughput expression profiling using unique molecular identifiers. Considering the variable range of CTCs in HCC^5^, our approach maximizes the odds of CTC detection. In this proof-of-principle study, we included 6 HCC patients and 1 control subject (Table 1). Four out of the six HCC patients had potential CTCs on IFC, as defined by a combination of factors: CD45 negative, at least 1 positive marker among pan-CK, GPC3, ASGPR1, or EPCAM, and compatible shape by bright field morphologic analysis. In accordance with previous reports^14^, the control subject had no candidate CTCs. As a pilot study, once IFC selection was optimized, we selected two out of the four patientswith candidate CTCs, those with higher number of CD45 negatively enriched cells, for further analysis with scRNAseq (Fig. 2A,B).

**Table 1:** Clinical characteristics and Imagestream analysis of the patients analyzed.

Patient	Gender	Age	Etiology	Cirrhosis	BCLC stage	AFP	Albumin (g/dl)	Bilirubin (mg/dl)	INR	Platelet (10E9/L)	Imagestream data					scRNAseq
											CTC count	EPCAM+	CK+	ASGPR1+	GPC3+	
Patient 1	M	68	HCV	Yes	C	448	2.7	1.4	1	209	5	5	3	2	0	Yes
Patient 2	M	77	HCV/Alcohol	Yes	C	460	1.9	1.8	1.2	116	5	5	5	0	0	Yes
Patient 3	M	64	Cryptogenic	No	C	140	3.2	1.1	1	200	11	11	11	4	0	No
Patient 4	M	59	HCV/Alcohol	Yes	B	26	3.4	0.8	1	116	0	0	0	0	0	No
Patient 5	M	63	HCV	No	A	2	3.5	0.2	1.1	342	4	4	0	0	0	No
Patient 6	M	44	HBV	Yes	A	1	3.6	0.5	1.2	114	0	0	0	0	0	No
Patient 7*	F	56	—	No	NA	NA	4.2	0.6	1	222	0	0	0	0	0	No

*Subject without cancer; NA: Not applicable; HCV: Hepatitis C virus; HBV: Hepatitis B virus; M: Male; F: Female; AFP: Alpha-fetoprotein; BCLC: Barcelona Clinic Liver Cancer.

**Figure 3:**
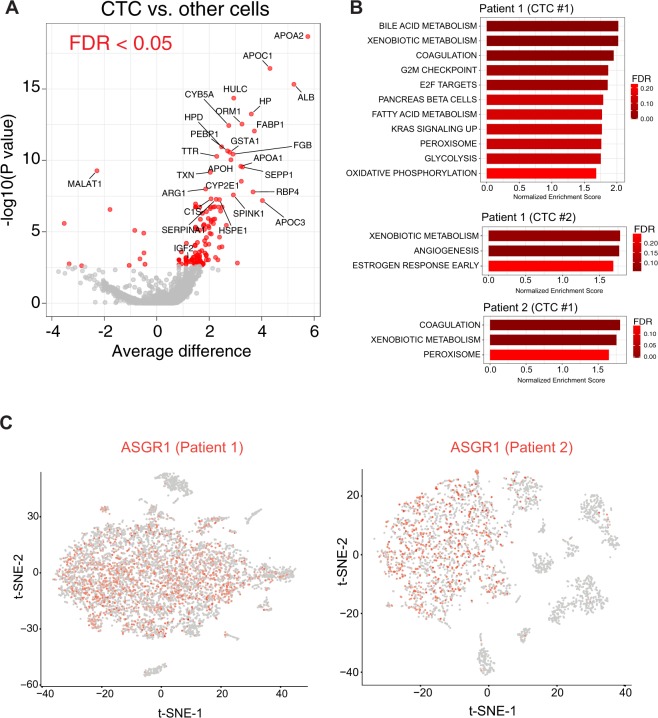
Characterization of CTCs and identification of potential HCC driver genes. (**A**) Volcano plot of differential gene expression between the 3 CTCs found in the 2 patients and the rest of blood cells. Red dots denote comparisons with an FDR < 0.05. (**B**) Bar size represent normalized enrichment score values and FDR (i.e., red gradient) for gene sets significantly enriched in each CTC resulting from GSEA. (**C**) Expression of ASGR1 on scRNAseq in non-CTC blood cells. Scaled expression depicted as a red gradient (grey denotes no expression detected for ASGR1).

Due to the rarity of CTCs in the blood, we adjusted the analytical scRNAseq pipeline to be specifically sensitive to detect outliers. We sequenced a total of 7,104 and 3,130 cells in patients 1 and 2, respectively. Median number of RNA molecules and genes detected per cell in patient 1 were 3,046 (range 1,004–21,169) and 1,098 (range 202–4,109). In patient 2, we detected a median of 2,552 RNA molecules (range 1,147–27,950) and 933 genes (range 202–4,484) per cell. We used the most variable genes to compute the main sources of variability in the dataset, as depicted in a principal component (PC) analysis (Fig. 2C,D). This approach identified two CTCs from patient 1 and one CTC from patient 2 with gene expression profiles dramatically different from the other clonal populations in the blood. These candidate CTCs aligned along the first PC, emphasizing the strong source of variation captured by this PC. In both cases, the genes that defined PC1, which captures the maximum variance, were highly weighted on liver specific genes (STable 1). As predicted, when comparing the scaled expression values of the 3 candidate CTCs from the 2 patients with the rest of the cells, we also found numerous liver-related genes among the 100 top differentially expressed genes (Fig. 3A and STable 2). These genes are involved in liver physiology including apolipoproteins, coagulation factors, alcohol dehydrogenases, cytochromes, etc. The 3 candidate CTCs isolated from both patients shared 12% of their top 100 expressed genes (*TTR*, *FABP1*, *GSTA1*, *APOH*, *FGB*, *HULC*, *ALB*, *APOA2*, *SEPP1*, *APOC1*, *HPD* and *ORM1*), which increased our confidence of efficient CTC detection. To further support a hepatic lineage of these outlier cells, we performed gene set enrichment analysis (GSEA) on their scaled expression as compared to all the other cells. Bile acid and xenobiotic metabolism were among the top positively enriched gene sets in these cells (Fig. 3B). We next sought to characterize the rest of the blood cells based on their gene expression profiles. Using the marker genes that define each of these clusters (Fig. 2C,D and STable 3) we identified cells compatible with monocytes, NK cells, B and T cells, plasmacytoid dendritic cells and red blood cells.

As predicted, our CD45 negative selection increased the ratio of CTCs to non-CTCs up to approximately 1 CTC per ~3,000 nucleated cells. In this context, high-density scRNA-seq is essential to accurately discriminate CTCs using whole transcriptome data. Unexpectedly, when we checked the expression of ASGPR1, a marker used to detect CTC in HCC^15^, we found that it was expressed in a significant proportion of non-CTCs, mainly monocytes (Fig. 3C). Non-hepatic expression of ASGPR1 has been recently described^16^, which further underscores the added value of genome wide scRNAseq for accurate CTC detection. Altogether, these observations provide further evidence of the limitations of single (or targeted) marker approaches for CTC detection. In summary, scRNAseq detected cells in the blood of HCC patients with a genomic profile highly suggestive of an hepatic lineage.

### scRNAseq identifies HCC driver genes and suggest molecular heterogeneity in CTCs

Single-cell RNAseq was very suggestive of a hepatic origin of the outlier cells, but the low-coverage and sparse nature of these data precluded an evaluation of unequivocally malignant traits such as somatic mutations or chromosomal aberrations. Sequencing depth was suboptimal to confidently call mutations in well-known HCC hotspots. However, in both patients, candidate CTCs expressed Hepatocellular Carcinoma Up-Regulated Long Non-Coding RNA (HULC), while absent in the other blood cells. HULC is a lncRNA with known oncogenic properties in HCC^17^. We also detected other genes frequently up-regulated in HCC such as SPINK1, IGF2, SPP1 or BRIC5 (STable 2). This, added to the fact that circulating cells of hepatic lineage are neither detected in the bloodstream of healthy donors nor in patients with non-malignant chronic liver diseases^13^, supports the malignant nature of our candidate CTCs.

Despite the mutational landscape of HCC is fairly known^18^, the most prevalent HCC mutations are undruggable. Indeed, HCC ranks second to last in terms of number of druggable mutations^19^, which emphasizes the need to develop new approaches to detect druggable events in HCC patients. Among the differentially expressed genes in our CTCs, we detected overexpression of IGF2 (Fig. 3A and STable 2), a gene overexpressed in 20% of early HCCs and recently characterized as an HCC epidriver^20^. Indeed, selective blockade of IGF2 using monoclonal antibodies has shown anti-tumoral effects in experimental models of HCC. Notably, IGF2 de-regulation cannot be assessed by merely probing the mutation landscape of the tumor, which renders scRNAseq of CTCs an outstanding tool to detect non-mutated druggable genomic aberrations such as IGF2 overexpression.

In addition to IGF2, many genes were differentially expressed between the two candidate CTCs detected in patient 1, as captured by their distribution along PC1. Also, GSEA revealed differences in the top ranked gene sets enriched in the 2 candidate CTCs found in this patient, including features suggestive of a more aggressive biological behavior in CTC #1 (e.g., E2F targets, G2M checkpoint, KRAS signaling, Fig. 3B). These data are indicative of transcriptome heterogeneity in the CTC compartment, and adds credence to our hypothesis that high-density expression profiling using scRNAseq could help trace cancer heterogeneity in CTCs. Unfortunately, these patients had advanced HCC and were diagnosed using imaging techniques as per clinical practice guidelines, which prevented us to access tumor tissue to recapitulate our findings on CTCs in corresponding tissue. In summary, we have applied scRNAseq to identify and characterize CTCs in patients with HCC. Our proof-of-principle study demonstrates the advantages of genome-wide transcriptome profiling to confidently detect CTCs, its potential role to monitor HCC heterogeneity and its ability to detect HCC driver genes, which will ultimately help customize therapeutic interventions in these patients.

## Experimental Procedures

### Human samples, cell sorting and imaging flow cytometry

All patients were enrolled at a single institution (Icahn School of Medicine at Mount Sinai (ISMMS)).The study was approved by the ISMMS Program for Protection of Human Subjects/Institutional Review Board with protocol number HS-15-00540. All patients provided informed consent to participate in this study. All methods were performed in accordance with the relevant guidelines and regulations for conducting research in human subjects. Patients were diagnosed with hepatocellular carcinoma (HCC) based on the EASL guidelines (3), except one who was enrolled as a control subject (i.e., no tumor disease). Blood samples were collected in 8 ml heparin tubes (Vacutainer®, BD Bioscience) and processed within 6 hours after collection. Depletion of haematopoietic cells (red cells and CD45-positive cells) was performed by an immunodensity assay (Rosette Depletion Kit®, Stem Cells and Ficoll-Paque Plus, GE Healthcare). The enriched cell layer at the interphase between plasma and the density medium was collected and washed with 2%FBS-PBS for platelet depletion by centrifugation according to the manufacturer. Vital cells were counted with a Neubauer haemocytometer and trypan blue staining and resuspended in 2% FBS-PBS. To account for red cell contamination, a red cell lysis step was performed (Red cell lysis buffer®, eBioscience). One aliquot of 10,000 cells was collected and stored on ice. The remaining cells were stained with the following conjugated antibodies ASGPR-1-FITC (sc-52623 FITC; Santacruz Biotech), pan-CK-PE (Clone-C11, 10478; Cayman Chemicals), Glypican-3-APC (FAB2119A; R&D systems), EPCAM-VioBlue (CD326(EpCAM)VioBlue; Mylteni), CD45-Cy5-PE (PE-Cy™7 Mouse Anti-Human CD45; BD Pharmingen) and analyzed by Imaging Flow Cytometry (IFC, Imagestream X Amnis, Millipore) following a similar approach as previously described^14^. Of the twelve channels available, channel 1 was reserved for bright field images, whereas channels 2, 3, 6, 7 and 11 were used for the detection of the fluorochromes. Compensation beads were used to provide single color reference samples for each fluorochrome. A compensation matrix was built with these reference samples to remove the spectral overlap to adjacent channels from each detection channel. CD45-negative cells showing positivity for at least 1 marker of our antigen panel (i.e., ASGPR1, pan-CK, GPC3, EPCAM) and with a bright field image allowing the identification of their morphology were defined as potential CTC.

### Single cell RNA sequencing

For those samples with potential CTCs based on Imagestream analysis, the second aliquot was immediately used for single-cell RNA sequencing (scRNAseq) using Chromium (10X Genomics) and Illumina NGS sequencing technologies. The cell density was determined using a C-Chip DHC-N01 disposable hemocytometer. A mixture of 5 μl of 0.04% trypan blue (BioRad) and 5 μl of the single cell suspension was loaded onto the hemocytometer and examined at 10X magnification, using the Invitrogen Evos FL Cell Imaging System-digital inverted microscope. The single-cell chip loading, Gel bead in Emulsion (GEM) generation & barcoding, post GEM-RT & cDNA amplification, and library construction were performed according to the Chromium^TM^ Single Cell 3′ Protocol - Chemistry v2. For GEM generation an input of 10,000 cells total, at 1,000 cells/uLdensity, was targeted for each sample, with a target cell recovery of 6,000 cells. Library construction, enzymatic fragmentation, End-repair and A-tailing were performed as follows: pre-cool block at 4 °C hold, fragmentation at 32 °C for 5 minutes; End repair and A-tailing 65 °C for 30 minutes and held at 4 °C. Post reaction cleanup was performed, followed by adaptor ligation. Adaptor ligation incubation was done at 20 °C for 15 minutes. Post adaptor ligation cleanup was then performed, followed by sample index PCR with the following parameters: 98 °C for 45 seconds; followed by 14 cycles: 98 °C for 20 seconds; 54 °C for 30 seconds; and 72 °C for 20 seconds; followed by 72 °C for 1 minute and held at 4 °C. Quantification of the constructed libraries was evaluated using QubitdsDNA HS Assay Kit (Thermo Fisher), Agilent cDNA High Sensitivity Kit, and Kapa DNA Quantification Kit for Illumina platforms, following the manufacturer’s instructions. Generated libraries were sequenced on the Illumina HiSeq. 2500, using the paired-end 2 × 125 bp sequencing protocol. Sequencing run parameters were set up according to version 2 chemistry, the number of cycles for each read as follows: Read 1: 26 cycles, i7 index: 8 cycles, i5 index: 0 cycles and Read 2: 98 cycles.

### Data analyses

We used the package Seurat^21^ for data analysis using several adjustments to specifically increase the sensitivity to outliers. These tuned parameters and filtering involve the consideration of all genes as input regardless of the percentage of cells expressing them. No upper-bound limits were enforced when scaling the genes. The dataset was analyzed adjusting for the effect of the total amount of expression, or unique molecular identifiers (nUMIs), and the percentage of mitochondrial reads by means of linear regression. Subsequently, a Principal Component Analysis (PCA) was performed using only the genes identified as highly variable (1,223 and 1,054 for patient 1 and 2 respectively) for computing the principal components. These highly variable genes are selected among those with the highest variation among each of the thirty bins in which the genes are categorized according to their normalized strength of expression. No lower limits regarding strength of expression were imposed in order to capture the variation derived from the subtle expression signal of CTCs. Subsequently, we visualized this data in a two-dimensional PCA space to identify the outliers. The marker genes for these candidate CTCs were manually curated, and GSEA was run on scaled fold-changes of each CTC vs. all others cells. The loadings of the principal component (PC) 1 rank the genes that more accurately discriminate all the elements along this PC. As a proxy for expression differences in individual CTCs versus non tumoral cells, and given the limitations for differential expression testing in individual samples (dropout and lack of replicates to estimate variance), we used the residuals of expression after normalization (i.e., scaling, centering) and regression.

For the analysis of the rest of the blood cells we used a robust method with upper-limit scaling followed by the linear regression of the undesired effect of nUMIs and percentage of mitochondrial reads. This analysis was less contingent on outlier detection, so we followed a conventional approach using non-linear clustering, filtering out those genes which are expressed in less than 5 cells, and capping scaled maximum expression. Next, we performed dimensionality reduction with PCA on all genes, and later we clustered the cells with a graph-based algorithm for modularity optimization on principal component space. We added to this robust clustering method the supervised labeling of the outliers identified by the outlier-focused analysis (i.e., the CTCs. See above). After this, we computed marker genes^22^ for the blood cell clusters modules (not CTCs). Subsequently we applied manifold learning techniques (non-linear dimensionality reduction) to gain insight on the structure of the data and clusters. In particular, we used tSNE^23^ on PC space and used it to visualize the clusters in the blood.

In order to gain power to calculate marker genes for each cluster, we performed a combined analysis of the two single cell datasets of both patients. For the combined analysis, we selected the union of the top 2,000 genes with the highest dispersion (variance to mean ratio) from both datasets. We then ran a canonical correlation analysis (CCA) to identify common sources of variation between the two datasets. The result is, effectively, a form of dimensionality reduction, where 20 canonical vectors were calculated. The distributions of the cells’ embeddings for these canonical vectors were aligned in order to remove batch effects (i.e. aligning the CCA subspaces). Next, on the combined dataset, we ran maker selection algorithms; first using Area Under the ROC Curve to rank the genes with most predictive power for each cluster. We used as well a bimodal (discrete and continuous) test^22^ for single cell differential expression. Analyses were performed using R and raw sequencing data are deposited in NCBI’s Gene Expression Omnibus under accession number GSE107747.

## Electronic supplementary material

Supplementary tables
